# CT analysis of frontal recess air cell and fluid dynamics simulation of frontal sinus in people with different frontal sinus development after Draf1–3 surgery

**DOI:** 10.1007/s00405-023-08433-8

**Published:** 2024-01-08

**Authors:** Zhengru Zhu, Jian Wang, Weijia Du, Min Xu, Tao Xue, Yubing Lai, Fuquan Chen

**Affiliations:** Department of Otorhinolaryngology Head and Neck Surgery, Xijing Hospital, Air Force Medical University, Fourth Military Medical University, 127 West Changle Road, Xi’an, 710032 Shaanxi China

**Keywords:** Frontal sinus development, Computational fluid dynamics (CFD), Three dimensional reconstruction, Sinus airflow, Sinus irrigation

## Abstract

**Objective:**

To explore the effects of Draf1–3 on frontal sinus airflow and frontal sinus irrigation in people with different frontal sinus development

**Methods:**

The development of the frontal sinus and the distribution of the frontal recess cells were evaluated by CT scan in 150 adults (300 sides). The airflow changes into the frontal sinus and frontal recess after Draf were analyzed by Fluent software under a steady state and quiet inspiratory state. Nasal irrigation after Draf in adults with well-developed frontal sinus was simulated using 120 mL saline at a rate of 12 mL/s in a position at 45° to observe the changes in transient flow distribution.

**Results:**

The moderately developed type of the frontal sinus was the most common. The airflow patterns in the frontal sinus and frontal recess in the moderate development group were laminar, while several large vortexes were formed between the frontal sinus and frontal recess in the well-development group. The Draf exerted more significant effects on the patterns, pressure, and velocity of the airflow in the frontal sinus and frontal recess in the well development group than in the moderate development group. The volume fraction of saline in the frontal sinus increased significantly from Draf1 to Draf3, and the time required for a complete infiltration of saline in the frontal sinus mucosa was significantly reduced.

**Conclusions:**

Draf1–3 has different effects on the airflow field of the frontal sinus with different developmental types; and Draf1–3 can significantly improve the postoperative flushing of the frontal sinus.

## Introduction

The anatomical structure of frontal sinus and frontal recess is complex and variable, making difficult to visually expose the frontal sinus ostium and frontal sinus drainage channel, and becoming a more challenging area in endoscopic sinus surgery. In 2017, Comoglu et al. divided the development of frontal sinus into four types [5]. In 2016, Wormald et al. proposed The International Frontal Sinus Anatomy Classification (IFAC) [21]. This article provides a more precise and systematic nomenclature for the air cells around the frontal recess. The purpose of this study is to provide the imaging data of the development of the frontal sinus and the air cells around the frontal recess under the IFAC classification in normal Chinese adults, to propose a theoretical basis for the simulation of the flow through the frontal sinus after Draf surgery with different types of frontal sinuses. In 1991, Wolfgang Draf classified the endoscopic frontal sinus surgery into Draf1 (Remove the obstructive lesions below the frontal sinus ostium and remove the anterior ethmoid cells, mainly the agger nasi cells, which blocks the frontal sinus drainage channel. The operation does not involve the frontal sinus ostium), Draf2a (Based on draf I, the floor of the frontal sinus between the middle turbinate and the orbital cardboard and the ethmoid cells invading the frontal sinus were removed to locally enlarge the frontal sinus ostium and form a channel between the middle turbinate and the orbital cardboard. The root of the middle turbinate was not involved in the operation), Draf2b (Based on draf IIa, the root of the middle turbinate was excised inwardly so that the medial margin of the frontal sinus opening reached the nasal septum)and Draf3 (On the basis of bilateral draf II, the floor wall of the bilateral frontal sinus, the adjacent upper nasal septum, and the septum of the frontal sinus were excised, and bilateral penetrating drainage channels were established)according to the degree of frontal sinus lesions and the extent of surgical resection [19]. This is currently the most widely used classification of the frontal sinus surgery worldwide, but going under continuous improvements. In 2013, Komser et al. proposed a new naming method such as Draf2c (On the basis of draf IIb, the middle frontal sinus septum and the upper nasal septum were removed), in order to minimize the surgical operation of Draf3 on non-diseased areas [2]. In 2016, the International Classification of frontal sinus endoscopic surgery was further refined and divided into seven grades, although at present, Draf classification is the most commonly used in clinical practice [15]. In recent years, computational fluid dynamics technology has been widely used in medicine. In our field of rhinology, it is mainly used to simulate the normal state, various pathological states, and the changes of nasal cavity and paranasal sinuses before and after surgery. At present, few studies are available on the distribution, pressure, nasal resistance, and velocity of the airflow in the frontal sinus after Draf surgery. Previous studies suggested that the airflow in the normal frontal sinus is very small and can be ignored, but the airflow into the frontal sinus after Draf surgery cannot be ignored. Li et al. found significant statistical differences in pressure and flow velocity between Draf III and the normal model, with the "frontal sinus T" structure as an important cause of edema after Draf III [11]. In this study, computer fluid dynamics (CFD) was used to simulate Draf1–3 in patients with different development of frontal sinuses, and the airflow characteristics of the frontal sinus before and after Draf were compared to further explore the influence of Draf on the airflow in frontal sinuses with different development. This provide a certain reference in the design of preoperative surgical plan and the evaluation of postoperative complications. Saline nasal irrigation has been added into the guidelines as a routine treatment for patients with chronic rhinosinusitis after surgery. Early postoperative nasal irrigation can significantly prevent nasal crust and nasal adhesion, improving the course of that treatment that is at least four weeks. Saline irrigation can not only remove inflammatory factors from the nasal cavity and promote mucosal secretion of mucin, but also improve the ciliary clearance function and reduce mucosal congestion [7]. However, few studies are available on the hydrodynamic changes brought by saline through the nasal cavity and paranasal sinuses before and after Draf surgery, especially the changes in the flow velocity, volume, and pressure of saline over time, and the changes in the fluid at different positions of the nasal cavity and paranasal sinuses. This study used CFD to simulate the process of saline irrigation before and after Draf, and the changes of transient water flow distribution in nasal cavity and paranasal sinuses were observed to provide a certain reference for the selection of the best position for nasal irrigation after frontal sinus surgery, the control of water volume and flow rate in the device used to product the nasal irrigation, and postoperative nasal drug delivery.

## Materials and methods

### Objects of the study

A total of 150 cases (81 males and 69 females) of sinus thin-layer CT scan data were selected from all CT scans performed in the Department of Radiology of Xijing Hospital from January 2010 to September 2017 according to the inclusion and exclusion criteria. The mean age was 40.08 years (18–74 years). A total of 300 paranasal sinuses were used for comparative analysis. The inclusion criteria were the following: adults over 18 years of age with consecutive CT scan of paranasal sinuses; the continuous and fine axial images can be used for three-plane fitting reconstruction. No enhancement was used in the CT scan. The exclusion criteria were the following: slice thickness > 2 mm, difficulty in completing three-dimensional plane reconstruction of fine structures, evident thickening of the nasal mucosa, sinonasal tumors, previous trauma, congenital abnormalities, previous history of sinus surgery, or other nasal diseases.

### CT scanning

A 128-slice Siemens CT scanner was used to scan the nasal cavity and paranasal sinuses (main parameters: slice thickness 1 mm, pitch 0.6 mm, resolution: 512 × 512 pixels, bone window width: 1500 HU, window level: 450 HU, soft tissue window width: 350 HU, window level: 512 × 512 pixels). The axial, coronal, and sagittal reconstructed images were read and compared using RadiAnt DICOM Viewer 4.6.5.

### Classification of the frontal sinuses and counting of frontal recess cells

**Fig. 1:**
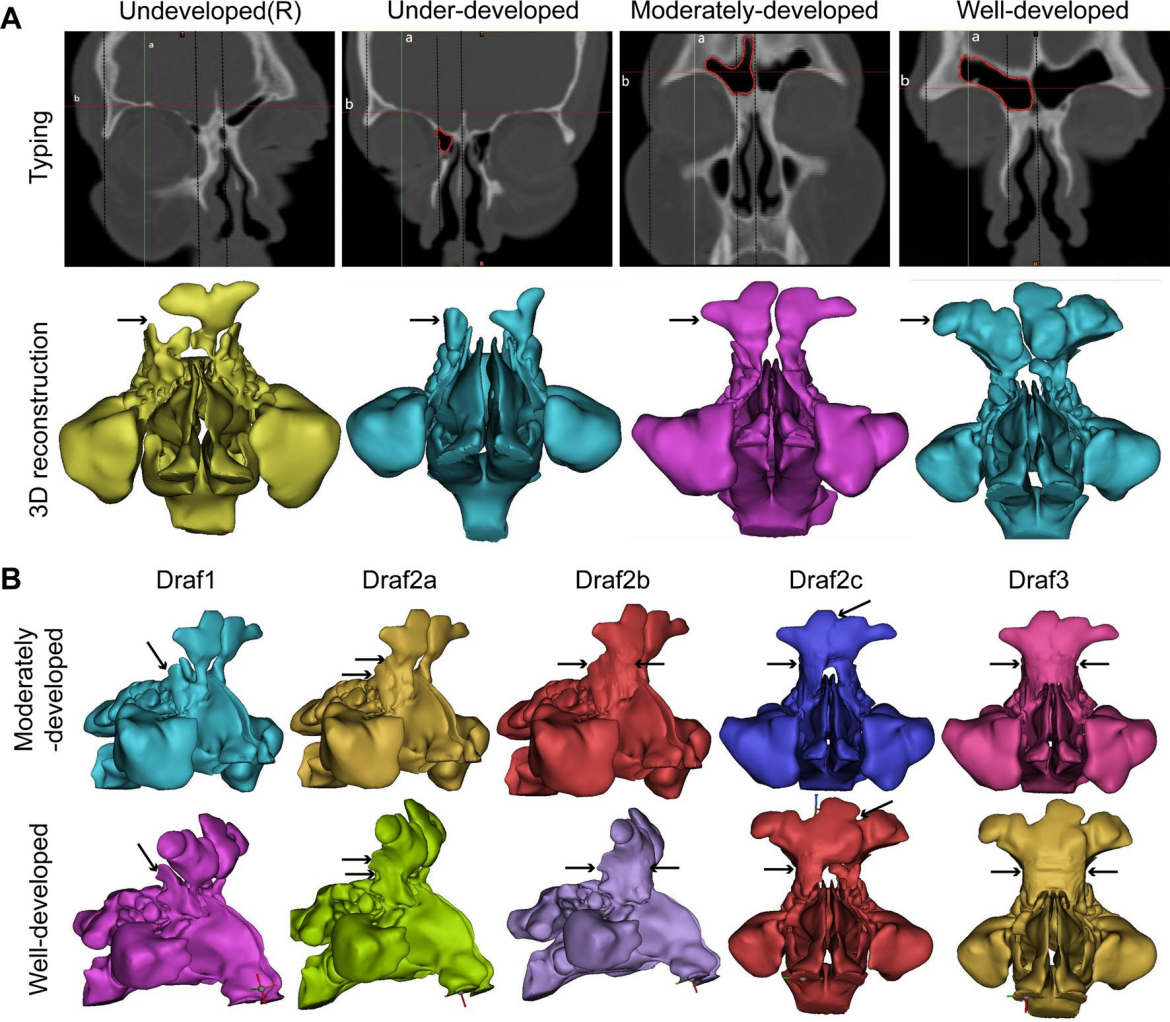
**A** Four types of frontal sinus development and three-dimensional reconstruction. **B** Three-dimensional reconstruction of the right Draf1–3 postoperative models

The horizontal line of the supraorbital margin in the coronal view was used as the transverse line (line b), and the vertical line was made at the supraorbital (neurovascular bundle) notch (line a). The development status of the frontal sinus was classified by the four-quadrant method [5]. The pneumatization of the frontal sinus within the lower quadrant was considered as under-development. The pneumatization of the frontal sinus range from the upper to the inner quadrant was considered as moderate development. The type with pneumatization extending to the outer upper quadrant was considered as well developed. If no pneumatized frontal sinus was present in the frontal bone, it was considered as undeveloped type (Fig. 1A). According to the IFAC classification criteria [21], three otolaryngologists familiar with the IFAC system independently read all CT scans, evaluated the presence and number of air cells around the frontal recess, and calculated the incidence of each type of air cell. Responsible cells (RC) were defined as those whose extent exceeded 50% of the frontal recess and might affect the drainage of the frontal sinus when the film was continuously read in the coronal and sagittal views.

### Three-dimensional reconstruction

Four out of 100 adults were selected for 3-dimensional reconstruction (the anatomical classification of the frontal sinus was right undeveloped, bilateral under-developed, bilateral moderately developed, bilateral well developed), and the sinus CT scan was normal. Among them, the adults with moderately developed and well developed included the agger nasi cells, bulla cells, supra bulla cells, and supra bulla frontal cells. There was no history of nasal and paranasal sinus diseases, cheek dysplasia, surgery, and trauma. The CT data obtained were imported into the Materialise's Interactive Medical Image Control System (Mimics21.0, Leuven, Belgium) in DICOM format. The threshold -700HU to 3071HU in the "threshold" module was designed to distinguish the nasal cavity and paranasal sinus airway from the soft tissue as a whole. The "Modify" module was automatically combined with the manual editing of the mask and the target mask was selected and segmented through the "Dynamic Region Grow" module. Next, the 3D model was generated in the "calculate part". Finally, the nasal cavity and paranasal sinuses models of four developmental types and the nasal cavity and paranasal sinuses models simulating Draf1–3 postoperative nasal cavity and paranasal sinuses of the right frontal sinus were reconstructed (Fig. 1A, B).

### Surface generation and meshing

The 14 3D models reconstructed in Mimics21.0 were imported into Geomagic studio 12.0 software in STL format, and the models were optimized by denoising, smoothing, accurate surface remodeling, and fitting surface remodeling. The obtained results were imported into Fluent pre-processing software ICEM-CFD (Ansys, Canonsburg, PA) in IGES format, the geometric model boundaries were created, the closed surface was generated, and finally the finite element mesh was divided to control the total mesh number of approximately 2 million to 3 million. The mesh minimum cell was a tetrahedral cell with a volume greater than 0 and no negative volume mesh, which was exported in MSH format.

### Air flow simulation

The files in MSH format were imported into Fluent 16.0 software (Ansys, Canonsburg, PA) to simulate the airflow during the nasal inspiratory phase. The relevant parameters were set, in which the medium was air, the air density was 1.225 kg/m^3^, and the viscosity coefficient was 1.8 × 10^–5^ Pa s. The steady-state k-epsilon turbulence mode was used, the SIMPLE algorithm was used, and the Navier-Stoke equation of viscous fluid motion was applied for the numerical calculation. The inlet was the bilateral anterior nostril plane with a pressure of 1 standard atmosphere (*P* = 101 325 Pa), and the outlet was the nasopharynx with a default pressure of 0 Pa. According to the range of respiratory frequency and tidal volume in normal physiological state, the tidal volume of each breath was selected to be 600 mL, the breathing cycle was *T* = 3 s, that is, the ventilation volume was 12 L/min, the breathing time was the same, and the respiratory wave was sinusoidal curve. The area of the nasopharyngeal airway was obtained from the reconstructed model, and the average velocity of the nasopharyngeal airflow outlet was calculated. The calculated results were imported into CFD-POST software (Ansys, Canonsburg, PA) to be subjected to post-processing, and the point diagrams, flow diagrams, wall shear stress diagrams, velocity clouds and pressure clouds were intercepted at different levels of different models. The maximum flow velocity, maximum pressure, maximum wall shear stress, nasal mucosal area (cm^2^), nasal volume (mL), and nasal mucosal area-volume ratio (mm^−1^) were recorded.

### Simulation of nasal cavity and sinus irrigation

Saline irrigation simulation was performed using Fluent 16.0 software on a total of six well-developed models before and after Draf1–3, and the best position was selected: the 45 degree tilt position (the angle between the long axis of the head and the ground was 45°), and then 120 mL saline was simulated at a rate of 12 mL/s, from the right nostril into the nasal cavity and sinus for a duration of more than 10 s. Boundary setting: the right anterior nostril was the entrance, and the left anterior nostril was the only outlet. It was assumed that the oral cavity and nasopharyngeal cavity were closed, blocking the passage of saline and air, and the nasal mucosal wall was fixed and smooth. The distribution and movement of the interface between two immisciable fluids (air and brine) were predicted using the multiphase free surface method (i.e., volume of fluid VOF), with the choice of "coupled volume fraction" and "density difference (buoyance)" for the multiphase flow model, fluid surface tension of 71 dyne/cm, and earth gravity of 9.8 m/s^2^. Much less dense air rises and stays on top of the salt solution, as defined by gravity and density differences. The transient k-epsilon flow model was used to simulate the details of air and brine motion, taking into account the initial brine momentum applied by the rinse bottle. Both advection scheme and turbulence values were set to “high resolution”. The simulation employed a transient scheme using a second-order backward Euler method to accurately capture the full fluid motion during irrigation. An initial time step of 5e^−6^ s was set, and adaptive time steps were determined based on a target optimal number of iteration loops (3–5), with a minimum time step of 1e^−6^ s and a maximum time step of 0.01 s. Convergence criteria were established such that the root mean square (RMS) of the residue was less than 1e^−4^.

## Results

### Proportion of different frontal sinus development types

**Fig. 2:**
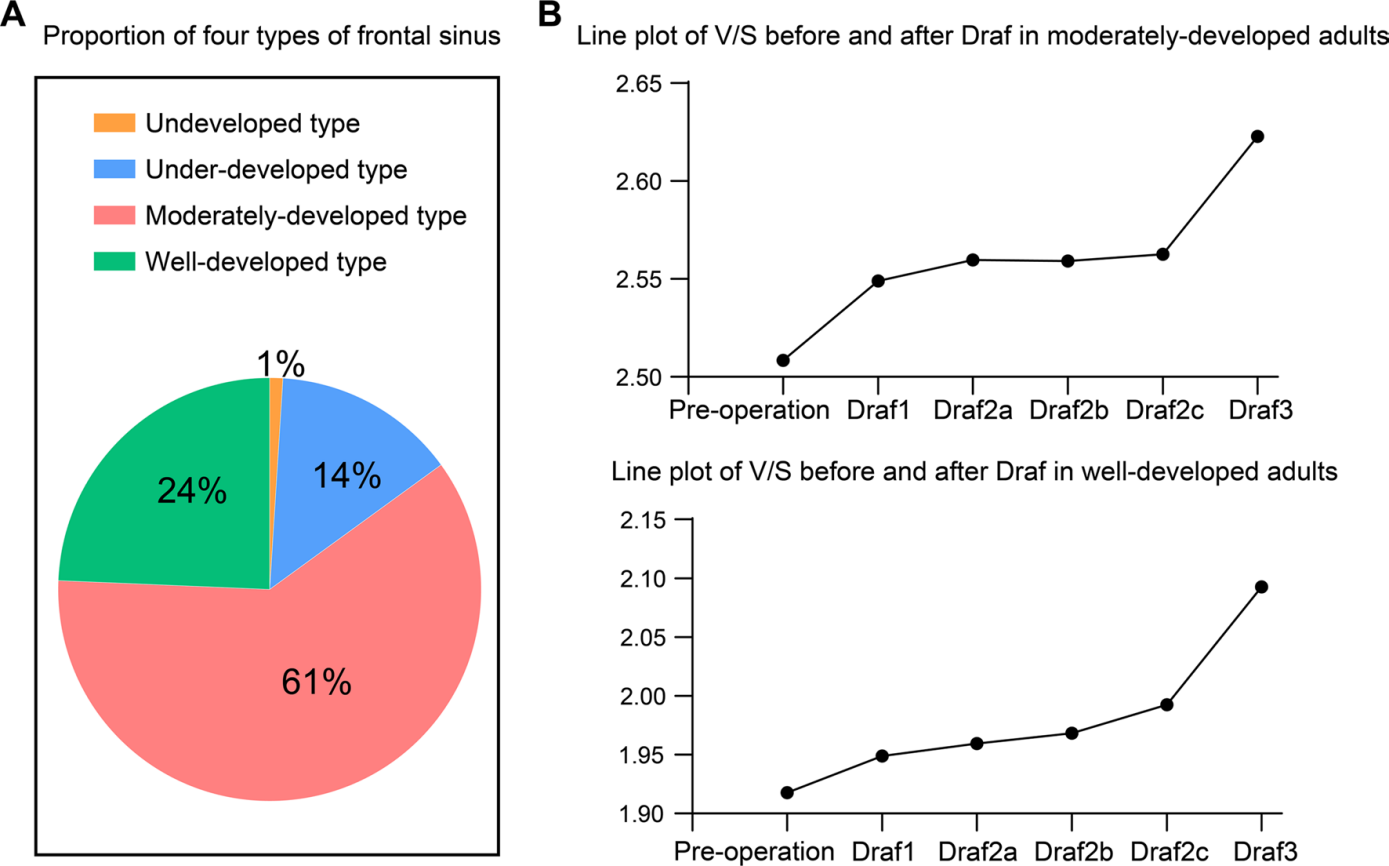
**A** Proportion of four types of frontal sinus. **B** V/S line plot before and after Draf in adults with different frontal sinus development

The CT data in this study revealed the presence of 3 cases of the undeveloped type (1 case on the left side and 2 cases on the right side); 42 cases of under-developed type (16 cases on the left side and 26 cases on the right side); 183 cases of moderately developed type (91 cases on the left side and 92 cases on the right side) and 72 cases of well developed type (42 cases on the left side and 30 cases on the right side). Among the total 150 cases, 35 cases (23.3%) had asymmetric frontal sinus development (Fig. 2A).

### Characteristics and ratio of frontal recess cells

A total of 259 cases had agger nasi cells (170 cases, 65.6%) and 99 cases had supra agger cells (78 cases, 78.8%), and among the former/latter cases, 9 cases had two supra agger cells. A total of 71 cases had supra agger frontal cells, all RC, including 3 cases with 2 supra agger frontal cells. A total of 281 cases had bulla cells (76 cases of RC, accounting for 27.1%), 206 cases had supra bulla cells (105 cases of RC, accounting for 51%), 107 cases had a single cell, 91 cases had 2 cells, and 8 cases had 3 or more cells. A total of 113 cases had supra bulla frontal cells (105 cases, accounting for 92.9%), including 104 cases having a single cell, 8 cases having 2 cells, and 1 case having 3 cells. A total of 112 cases had supraorbital ethmoidal cells (69 cases of RC, accounting for 61.6%), 68 cases had frontal septal cells, and 18 cases had RC, accounting for 26.5% (Table 1).

**Table 1:** Characteristics of the air cells around the frontal recess and their ratios

	Ratio of left cell and number of cases		Ratio of left responsible cell and number of cases		Ratio of right cell and number of cases		Ratio of right responsible cell and number of cases		Ratio of total cell and number of cases		Ratio of total responsible cell and number of cases	
ANC	85.3%	128	67.2%	86	87.3%	131	64.1%	84	86.3%	259	65.6%	170
SAC	34.0%	51	82.4%	42	32.0%	48	75.0%	36	33%	99	78.8%	78
SAFC	24.0%	36	100%	36	23.3%	35	100%	35	23.7%	71	100%	71
BC	94.0%	141	28.4%	40	93.3%	140	25.7%	36	93.7%	281	27.1%	76
SBC	68.0%	102	55.9%	57	69.3%	104	46.2%	48	68.7%	206	51%	105
SBFC	39.3%	59	93.2%	55	36.0%	54	92.6%	50	37.7%	113	92.9%	105
SOEC	36.0%	54	55.6%	30	38.7%	58	67.2%	39	37.3%	112	61.6%	69
FSC	22.7%	34	38.2%	13	22.7%	34	14.7%	5	22.7%	68	26.5%	18

*ANC* agger nasi cell, *SAC* supra agger cell, *SAFC* supra agger frontal cell, *BC* bulla cell, *SBC* supra bulla cell, *SBFC* supra bulla frontal cell, *SOEC* supraorbital ethmoidal cell, *FSC* frontal septal cell

### Changes in sinonasal volume and mucosal surface area after Draf1–3 surgery

The nasal mucosa surface area gradually decreased, the volume gradually increased, and the ratio of nasal ventilation volume to nasal mucosa surface area (V/S) gradually increased after Draf1–Draf3. Among them, the V/S after Draf2c–Draf3 significantly increased, and the increase of V/S after Draf1–Draf2c was small (Fig. 2B).

### Frontal sinus flow maps before and after Draf1–3 surgery in adults with different frontal sinus development

**Fig. 3:**
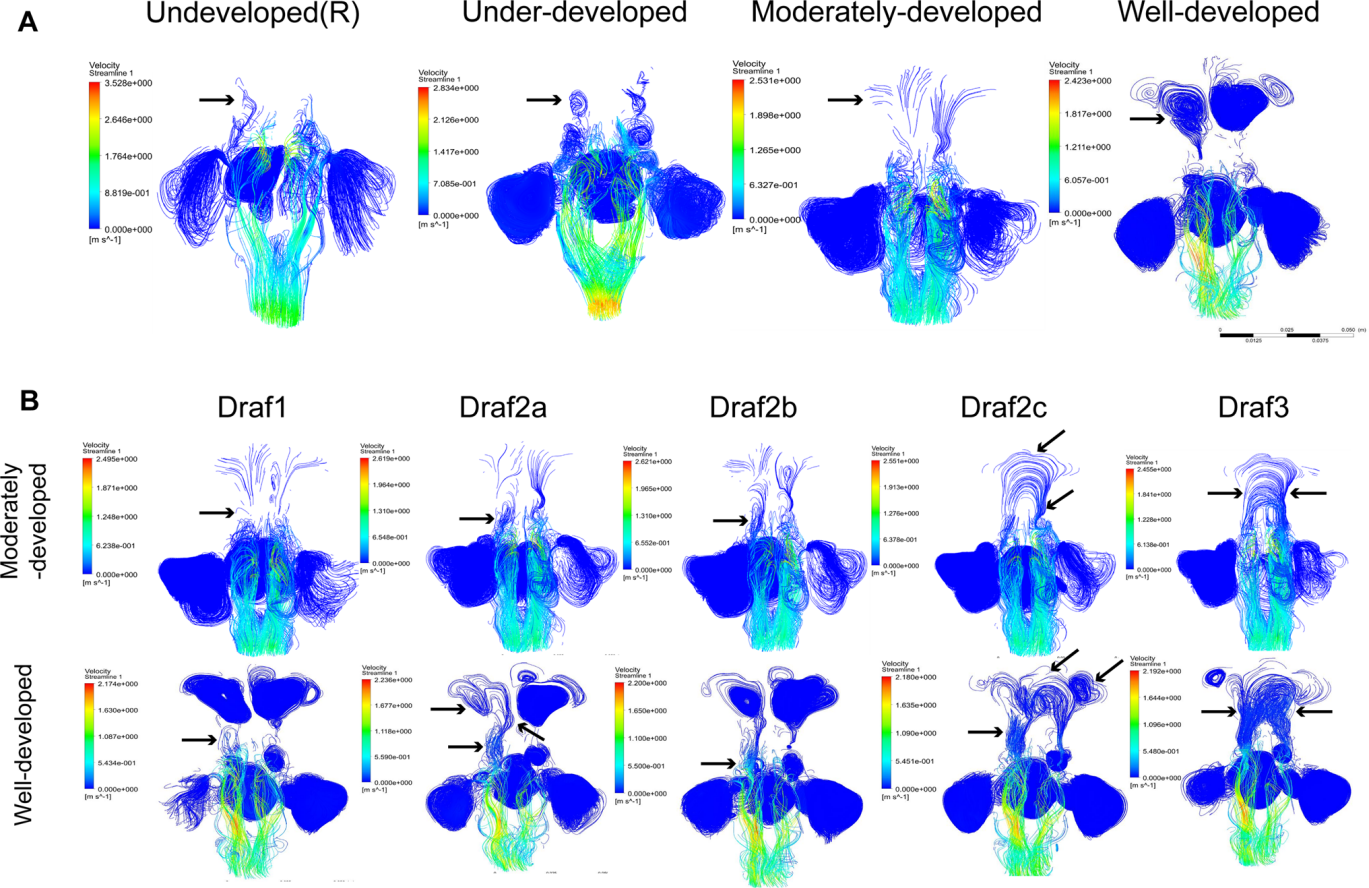
Frontal sinus flow maps before and after Draf1–3 surgery in adults with different frontal sinus development

The airflow in the nasal cavity during inspiration (Fig. 3A) was mainly distributed in the common meatus, followed by the middle meatus, the inferior meatus, and the olfactory cleft in the form of laminar flow, and the left and right were basically symmetrical. When the air flowed through the limen nasi, the flow velocity was at the maximum, and it decreased after it was divided into four strands. The flow velocity sharply increased when the four strands converged. The airflow in the frontal sinus gradually increased from the undeveloped to the well-developed frontal sinus. The airflow in the frontal sinus in the under-developed group was in the form of single vortex, the laminar flow in the moderately developed frontal sinus was in the form of laminar flow, and the airflow in the bilateral frontal sinus in the well-developed group was in the form of multiple turbulent flows. However, the airflow velocity in the frontal sinus, frontal recess cells, ethmoid sinus, maxillary sinus, and sphenoid sinus was close to 0. A small airflow inflow into the right frontal recess was observed after Draf1, but a very little airflow was found into the frontal sinus. A vortex was formed in the frontal recess area after Draf2a, and the airflow mainly entered from the medial side of the frontal sinus. Vortex flow was found in the lateral frontal sinus in the well-developed group, while laminar flow was found in the frontal sinus in the moderately developed group. Draf2b resulted in more airflow into the frontal recess than Draf2a after surgery, and the flow velocity at the base of the frontal recess was significantly larger in well-developed adults. The airflow in the bilateral frontal sinus was connected after Draf2c, and a larger vortex was formed in the right frontal recess area, with a larger flow velocity in the posterior wall of this area. A large vortex was formed in the middle of the bilateral frontal sinus in the well-developed group, and several small vortexes were observed at the edge. A laminar flow was still present in the bilateral frontal sinus in the moderately developed group, and a small amount of airflow in the left nasal cavity entered the frontal sinus. Several large eddities were formed in the bilateral frontal recess area after Draf3, with the maximum flow velocity and the most airflow going into the frontal sinus, especially in the well-developed group. Several large eddities were formed between the bilateral frontal sinus in the well-developed group, and large laminar flow was found in the bilateral frontal sinus in the moderate development group (Fig. 3B).

### The stress nephogram of frontal sinus before and after Draf1–3 surgery in adults with different frontal sinus development

**Fig. 4:**
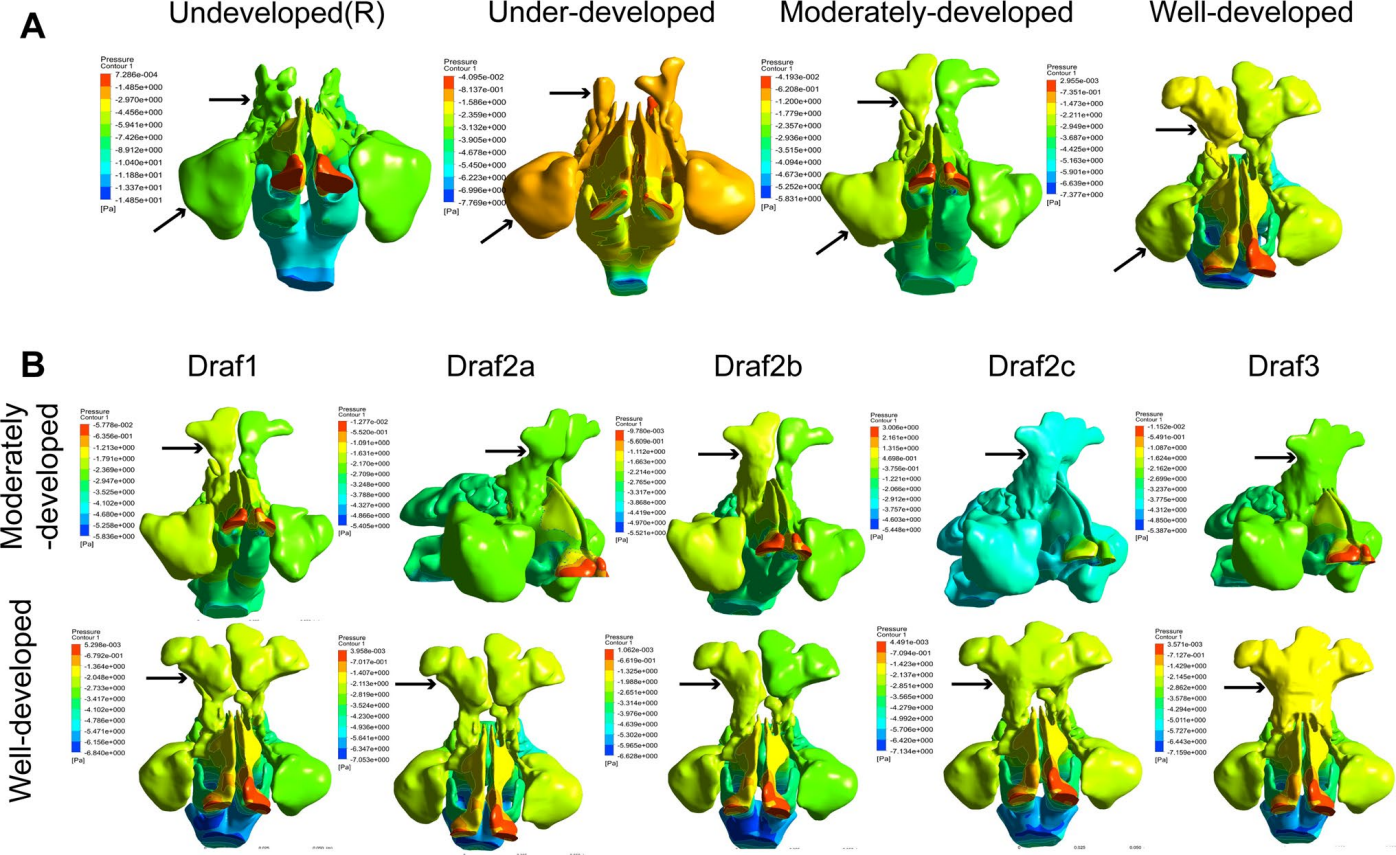
Frontal sinus pressure clouds before and after Draf1–3 surgery in adults with different frontal sinus development

The overall pressure of the nasal cavity and paranasal sinuses evenly and slowly decreased from the anterior nostril to the nasopharynx in normal subjects, and the pressure was the highest in the anterior nostril and nasal threshold. The pressure in the frontal sinus and maxillary sinus was similar, which was greater than in the ethmoid sinus; the pressure in the ethmoid sinus was greater than that in the sphenoid sinus, and the pressure in the sphenoid sinus was greater than that in the nasopharynx. Among the four developmental types, the pressure of the anterior ethmoid sinus was the lowest (− 7.426 to − 5.94 Pa) in the undeveloped people, the pressure in the frontal sinus and the maxillary sinus was the highest (− 1.586 to − 0.8137 Pa) in the under-developed people, and no significant difference was observed between the pressure in the frontal sinus and the maxillary sinus in the moderate and well developed people. No significant change in frontal sinus pressure was observed after Draf1–2b. The bilateral frontal sinus pressure significantly increased in adults with good development and significantly decreased in adults with moderate development after Draf2c-3 (Fig. 4A, B).

**Fig. 5:**
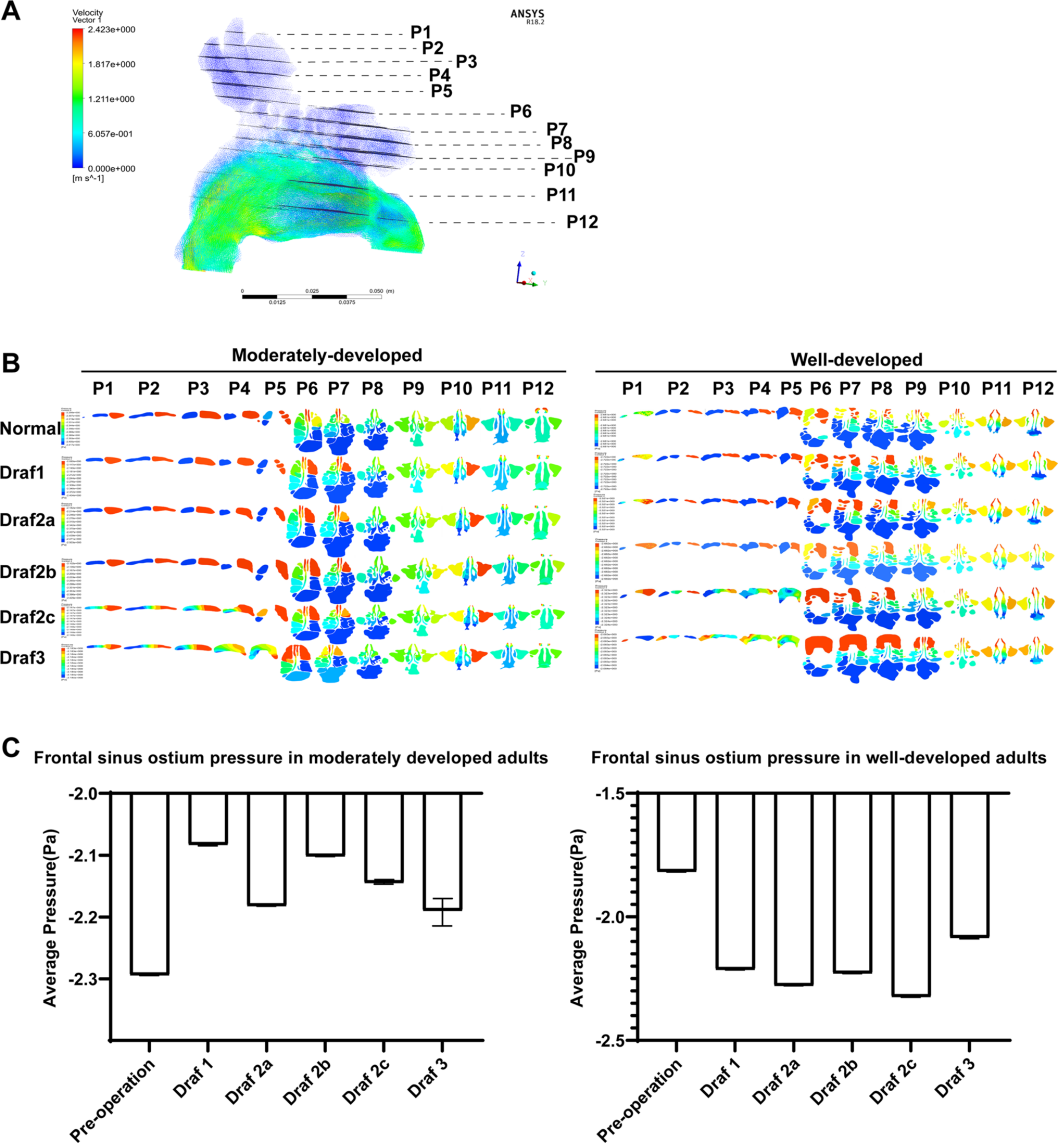
**A** Cross-section of plane 1–12. **B** Contours of pressure cross section after Draf in moderately developed and well-developed models. **C** Bar chart of average pressure in the frontal sinus ostium of moderately and well-developed individuals

Twelve horizontal sections (plane 1–12) were made from top to bottom along the longitudinal axis of the nasal cavity and paranasal sinuses, with an interval distance of 5 mm for plane 1–9 and 10 mm for plane 10–12 (Fig. 5A), and the local pressure maps of the 12 planes were captured (Fig. 5B). No significant difference in the pressure of the bilateral frontal sinus was found before operation, but it was not completely symmetrical, and the right side was slightly greater than the left side. On the same plane, the pressure of the bilateral anterior ethmoid was significantly higher than that of the posterior ethmoid, and the pressure of the olfactory cleft was significantly lower than that of the inferior meatus and common meatus. Draf1–2a had little effect on the pressure of the right frontal sinus and frontal sinus ostium, and mainly affected the anterior ethmoid pressure. The bilateral posterior ethmoid pressure also started to increase after Draf2b in adults with good development, but no significant change was observed in the adults with moderate development. The pressure in the right maxillary sinus gradually increases after Draf1–2b, which was significantly greater than that in the left. The pressure in the left frontal recess area also gradually increased in the well-developed subjects, but not in the moderate-developed subjects. The pressure in the right frontal sinus after Draf2c was greater than that in the left frontal sinus, and the pressure decreased from right to left in the same plane. The pressure in the frontal sinus ostium and frontal recess was the greatest, which was significantly greater than that in the common meatus and inferior meatus. The pressure in the frontal sinus decreased from right to left after Draf3 in the moderate development group, while it decreased from left to right in the well development group. The pressure in the frontal sinus ostium and frontal recess was the highest. The pressure in the bilateral maxillary sinus significantly increased. In addition, the bilateral olfactory cleft pressure slightly increased after Draf1–Draf3, and the mean pressure in the bilateral frontal sinus ostium did not significantly change (Fig. 5C).

### Contours of the frontal sinus velocity distribution before and after Draf1–3 surgery in adults with different frontal sinus development

**Fig. 6:**
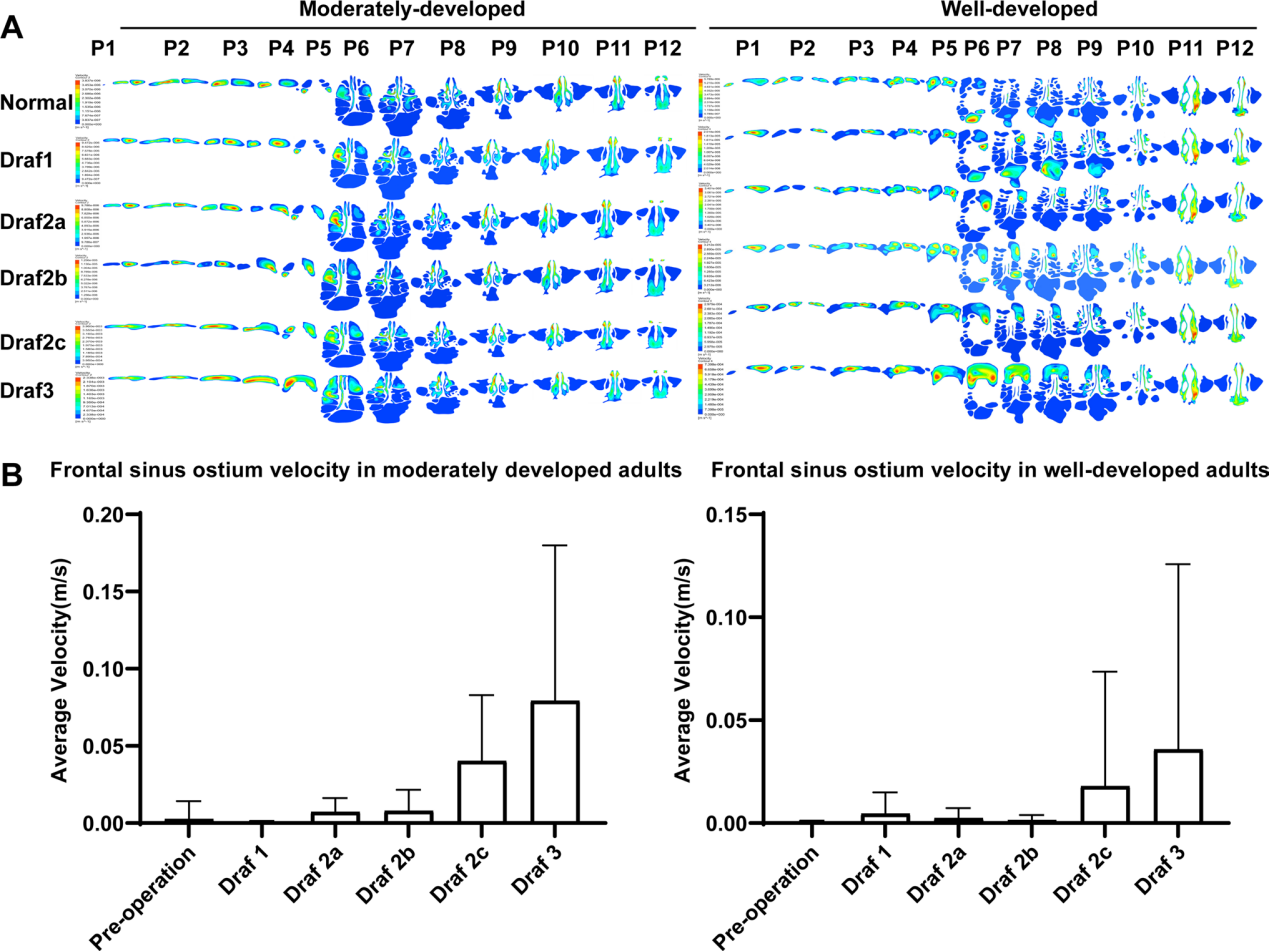
**A** Cross-section of velocity distribution after Draf in moderately and well-developed individuals. **B** Bar graph of mean velocity of frontal sinus ostium in moderate- and well-developed individuals

The airflow velocity of the nasal cavity and paranasal sinuses during inspiration before the operation (Fig. 6A) ranged from 0 to 5.789e−006 m/s, and the center of the maximum velocity in the bilateral frontal sinus was located in the center of the frontal sinus. The maximum flow velocity was found in the nasal threshold and the anterior part of the common meatus in the moderately developed group, and in the middle and posterior part of the bilateral common meatus in the well-developed group. A vortex was found at the top wall of the sphenoid sinus with a large velocity, and the velocity in the other sinus cavities was almost zero. The flow velocity in the right frontal sinus after Draf1 was significantly higher than that in the left side. A vortex was found in the frontal cell of the right ethmoid bubble; the flow velocity was significantly higher than that in the right frontal sinus, and the flow velocity in the middle of the sphenoid sinus increased. Multiple centers of maximum velocity were present in the right frontal sinus in the well-developed adults after Draf2a. A large vortex was found in the right frontal sinus ostium and the posterior wall of the frontal recess, and the central velocity was the largest. No significant change in airflow velocity was found after Draf2b compared with Draf2a. The center of the maximum flow velocity above the frontal sinus ostium after Draf2c was located in the center of the bilateral frontal sinus, below the frontal sinus ostium; the center of the maximum flow velocity was located in the posterior part of the right frontal recess. The maximum velocity center above the frontal sinus ostium gradually moved to the left from the center of the bilateral frontal sinus after Draf3. The maximum velocity center below the frontal sinus ostium appeared in the bilateral frontal recess area. In contrast, moderate developmental subjects had only one center of maximal velocity, which was fixed in the center of the frontal sinus. The flow velocity in the bilateral ethmoid sinus, sphenoid sinus, and maxillary sinus after Draf1–3 surgery was almost zero, and the effect on the flow velocity in the common meatus, middle and inferior meatus was also small. The average velocity in the frontal sinus ostia increased significantly after Draf1 to Draf3, and the maximum velocity difference was significant, especially in the middle development group (Fig. 6B).

### Instantaneous volume fraction of the nasal cavity and sinus irrigation after Draf in adults with well-developed frontal sinus

**Fig. 7:**
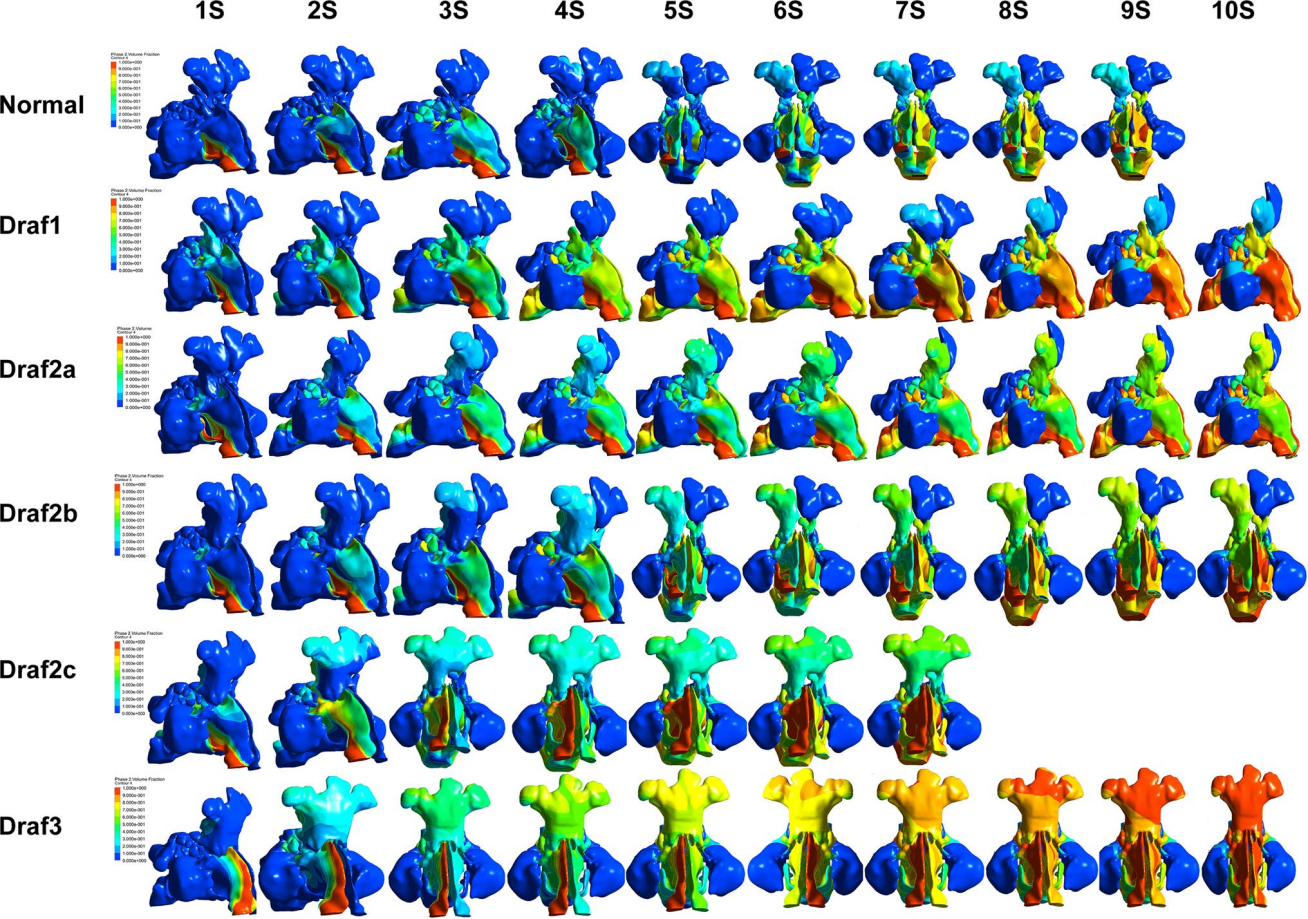
Instantaneous volume fraction map of nasal cavity and paranasal sinuses after Draf1–3 surgery

The water that was used to fill the nasal cavity before the operation, flowed through the bottom of the nasal cavity at the beginning, along the common nasal meatus, upward oblique to the bottom of the middle nasal meatus, and finally flowed to the contralateral nasal cavity through the top wall of the nasopharynx. The water flowed upward through the drainage channel of the frontal sinus through the opening of the frontal sinus at 4 s, and the inner posterior wall of the frontal sinus continued upward, and a small amount of water started to enter the frontal sinus. a small amount of water penetrated the whole frontal sinus at 7 s, a small amount of water entered the right anterior ethmoid, and almost no water entered the maxillary sinus, posterior ethmoid and sphenoid sinus. A small amount of water after Draf1started to enter the posteromedial wall of the frontal sinus at 6 s, and the water penetrated the entire frontal sinus at 9 s. The water after Draf2a started to enter the frontal sinus at 3 s, and infiltrated the entire frontal sinus at 5 s. The water after Draf2b started to enter the frontal sinus at 3 s, and infiltrated the entire frontal sinus at 4 s. The water flow after Draf2c entered the frontal sinus at 2 s and infiltrated the bilateral frontal sinus at 4 s. The water flow after Draf3 completely infiltrated the bilateral frontal sinus at 2 s. The water flow into the frontal sinus cavity after Draf1–3 gradually increased, and the volume of saline entering the sinus cavity reached the maximum after Draf3 (Fig. 7).

## Discussion

The frontal sinus is absent at birth, gradually develops with the development of the skull, and reaches its developmental peak in the adolescence. The development of the frontal sinus is greatly affected by the surrounding air chambers as well as by the inflammation of the nasal cavity and paranasal sinuses. The potential risk of stenosis of the frontal sinus drainage channel and frontal sinus retention inflammation caused by the variation of the frontal recess air chamber is higher than that of other sinuses. In our study, the most common type was moderately developed (61%), followed by the well-developed (24%) and hypoplastic type (14%), and the least was undeveloped (1%). In the study of Comoglu et al. [5], the most common type was the well-developed (49.1%), followed by the moderately developed (30.9%) and stunted (12%). The undeveloped type was the least (4%). In recent years, many experts in China and abroad have also carried out further anatomical, imaging, and clinical studies on the frontal recess cells. The agger nasi cells are relatively stable landmark cells. Previous studies showed that the recognition rate of the agger nasi cells is between 68.4 and 96.5%, and the recognition rate is approximately 90%. The recognition rate of the supra bulla cells and the supra bulla frontal cells is quite different; the former is between 11 and 90%, the latter is between 4.9 and 64.6%. The proportion of frontal septal cells is 4–33.1% [8–10]. The IFAC classification system is in line with the "building blocks" analysis method of the frontal sinus drainage channels previously proposed by Professor Wormald [20]. It is helpful to determine the direction of the frontal sinus drainage channels before the operation through a further classification and definition of the air cells around the frontal recess, and cells can be opened one by one according to the characteristics of the air cells during the operation. It is easy to locate the drainage channel of the frontal sinus, fully open the frontal recess and the frontal sinus and avoid the occurrence of orbital and intracranial complications. This study has important significance in guiding the surgery and reducing complications.

In recent years, the research of three-dimensional reconstruction and hydrodynamics in the nasal cavity and paranasal sinuses mainly focuses on three aspects: the simulation in the airflow changes of the normal human nasal cavity and paranasal sinuses, the simulation in the dynamics changes of the nasal cavity and paranasal sinuses fluid before and after surgery, and the simulation of the fluid changes after postoperative nasal irrigation and nasal spray administration. The earliest research was on the physiological changes of the airflow in the nasal cavity and paranasal sinuses of normal people. Since then, the airflow changes in the nasal cavity and paranasal sinuses before and after functional endoscopic sinus surgery have become a research hotspot in recent years, but mainly focusing on the overall airflow changes in the nasal cavity and paranasal sinuses as well as in the maxillary sinus. Previous studies have suggested that the airflow into the frontal sinus, ethmoid sinus, and sphenoid sinus in normal people during breathing can be ignored [3, 16, 18, 24]; thus, the description of the airflow characteristic in the frontal sinus is very limited. However, the airflow into the frontal sinus gradually increased with the gradual expansion of the surgical range of Draf1–3, and the airflow changes in the frontal sinus and frontal recess could not be ignored. In 2019, Li et al. found that the airflow pattern in the nasal cavity of the normal model was almost laminar [11], a vortex was not found in the dorsal nasal region, and almost no air entered the frontal sinus. The airflow pattern in the nasal cavity after Draf3 was similar to the normal model, but a large amount of air was found to flow into the frontal sinus; our research results are consistent with this evidence. In addition, they found that relatively high pressure areas mainly appeared in the posterior wall of the frontal sinus ostium, while in our study, high pressure areas mainly appeared in the two lateral walls of the frontal sinus. The laminar flow pattern after Draf1–Draf3 was always observed in the frontal sinus of the moderate developmental group, while the large vortex pattern was observed in the bilateral frontal sinus of the well development group. The flow velocity in the frontal recess area of the two developmental groups gradually increased, especially in the well development group. Previous studies showed that the temperature in the central region of the nasal cavity gradually decreases with the increase of the nasal flow velocity, and the temperature exchange only occurs on the side close to the mucosa in the laminar flow mode, while the exchange time between the air flow and the mucosa is prolonged in the turbulent flow mode [13, 14]. Therefore, our hypothesis is that the air temperature in the frontal sinus and frontal crypt would decrease and the humidity would increase after Draf. Most of them are well developed. In 2005, Lindemann et al. considered the presence of multiple vorticity in the whole nasal cavity and paranasal sinuses after radical resection [12], as well as nasal airflow disorder, decreased strength in the contact between air and nasal mucosal wall, and decreased ratio of nasal mucosal surface to nasal cavity volume. However, in our study, a little change was observed in the airflow in the nasal cavity and paranasal sinuses after Draf1–2b. Consistent with this, the airflow in the frontal sinus and frontal recess was significantly changed after Draf2c and Draf3. The ratio of nasal cavity and sinus volume to mucosal surface area (V/S) reflects the degree of airway stenosis [24]. This study found that Draf2c and Draf3 significantly improved the degree of airway stenosis. Zhou et al. believed that the airflow change in the upper part of the nasal cavity after Draf3 had little effect on the overall airflow in the nasal cavity [23], but airflow change in the lower part of the nasal cavity can significantly change the airflow distribution, and our study is basically consistent with it. In 2012, Abouali et al. found that nanoparticles and particles are easily inhaled into the sinus cavity by air flow after maxillary sinus opening as the flow rate increased, while almost no particles enter the maxillary sinus before surgery [1]. This is because airflow is the carrier of particles and nanoparticles, especially in the location of geometric shape mutation and airway direction mutation [6]. Therefore, our hypothesis is that Draf was beneficial to the delivery of drugs in the frontal sinus and frontal recess, but at the same time, it also increased the deposition of pollen and air pollution particles, causing nasal and sinus discomfort.

Saline irrigation has been widely recognized in the treatment of nasal cavity inflammation and paranasal sinuses inflammation after surgery, but few studies are available on the fluid dynamics of saline flow through the nasal cavity and paranasal sinuses, as well as even fewer studies on frontal sinus irrigation after Draf. This study found that Draf1–3 surgery significantly improved the flushing of the frontal sinus mucosa. When the right nasal cavity was irrigated with 120 mL saline at a rate of 12 mL/s at a 45° position, the right frontal sinus mucosa was completely infiltrated, which was consistent with the study of Zhao et al. [22]. In addition, they suggested that Draf3 may lead to a decrease in the coverage of saline in the maxillary sinus and ethmoid sinus, and revealed that this may be due to the removal of the posterior nasal septum after Draf3, leading to a premature outflow of most saline through the posterior nasal septum region. The study by Salati et al.also suggested that although a better access of the saline to the sinuses is achieved through functional endoscopic sinus surgery [17], the resection of the posterior septum may limit the penetration through the posterior group of the sinuses because saline may enter the contralateral nasal cavity and flow out of the nasal cavity in advance. Our study also found a significant increase in the volume fraction of saline in the frontal sinus after Draf1 to Draf3, and a significant reduction in the time required for a complete infiltration of the frontal sinus mucosa. Barham et al. found that Draf3 had the highest lavage rate (90.7%), followed by Draf2b (81.3%) and Draf2a (50.1%) by a study of 8 cadaveric specimens, and our study was consistent with it [4].

## Conclusions

This work revealed the existence of four types of frontal sinus through the investigation of the population, identified the air cells around the frontal recess, identified the air cells responsible for the main obstruction, and pointed out the key points and difficulties of the surgery. On this basis, three-dimensional CT images combined with fluent fluid dynamics software were used to quantify the airflow changes of the frontal sinus and frontal recess before and after Draf with different frontal sinus development types and simulated the changes of frontal sinus flushing after surgery. In addition, Draf1–3 surgery significantly improved the flushing of the frontal sinus mucosa, which was helpful to determine the optimal surgical scope according to the different development of frontal sinuses, and the frontal sinus and frontal recess air chamber were accurately opened during the operation. It is important to design the position, water volume, flow rate and the design of the irrigator according to the different surgical types after operation. The limitations of this study include: (1) The sample size was small; (2) We study the airflow during a stationary period. The normal nasal cavity has a nasal cycle, and the airflow during inspiration and respiration is different. (3) As a numerical simulation method, CFD has many confounding factors, such as boundary condition setting, mesh quality, model selection, etc. (4) In our study, the inner wall of the nasal cavity was set as a rigid wall, but in actual situations, nasal hair, vascular plexus, mucosal secretions, etc., all have certain effects on nasal airflow.

## Data Availability

All data, analytic methods, and study materials in details are available from the corresponding author upon request.
